# Adherence to antiretroviral therapy and treatment outcomes among conflict-affected and forcibly displaced populations: a systematic review

**DOI:** 10.1186/1752-1505-6-9

**Published:** 2012-10-31

**Authors:** Joshua B Mendelsohn, Marian Schilperoord, Paul Spiegel, David A Ross

**Affiliations:** 1 Department of Infectious Disease Epidemiology, London School of Hygiene and Tropical Medicine, MRC Tropical Epidemiology Group, London, UK; 2 Public Health and HIV Unit, United Nations High Commissioner for Refugees, Geneva, Switzerland

**Keywords:** Antiretroviral therapy, Treatment outcomes, Forced migration, Refugees, Conflict, Adherence, Systematic review

## Abstract

**Background:**

Optimal adherence to highly active antiretroviral therapy (HAART) is required to promote viral suppression and to prevent disease progression and mortality. Forcibly displaced and conflict-affected populations may face challenges succeeding on HAART. We performed a systematic review of the literature on adherence to HAART and treatment outcomes in these groups, including refugees and internally-displaced persons (IDPs), assessed the quality of the evidence and suggest a future research program.

**Methods:**

Medline, Embase, and Global Health databases for 1995–2011 were searched using the Ovid platform. A backward citation review of subsequent work that had cited the Ovid results was performed using the Web of Science database. ReliefWeb and Médecins Sans Frontières (MSF) websites were searched for additional grey literature.

**Results and conclusion:**

We screened 297 records and identified 17 reports covering 15 quantitative and two qualitative studies from 13 countries. Three-quarters (11/15) of the quantitative studies were retrospective studies based on chart review; five studies included <100 clients. Adherence or treatment outcomes were reported in resettled refugees, conflict-affected persons, internally-displaced persons (IDPs), and combinations of refugees, IDPs and other foreign-born persons. The reviewed reports showed promise for conflict-affected and forcibly-displaced populations; the range of optimal adherence prevalence reported was 87–99.5%. Treatment outcomes, measured using virological, immunological and mortality estimates, were good in relation to non-affected groups. Given the diversity of settings where forcibly-displaced and conflict-affected persons access ART, further studies on adherence and treatment outcomes are needed to support scale-up and provide evidence-based justifications for inclusion of these vulnerable groups in national treatment plans. Future studies and program evaluations should focus on systematic monitoring of adherence and treatment interruptions by using facility-based pharmacy records, understanding threats to optimal adherence and timely linkage to care throughout the displacement cycle, and testing interventions designed to support adherence and treatment outcomes in these settings.

## Introduction

It is widely accepted that for HIV-positive persons on highly active antiretroviral therapy (HAART), high levels of adherence to treatment regimens are essential for promoting viral suppression and preventing drug resistance. However, conflict-affected and forcibly displaced populations such as refugees and internally-displaced persons (IDPs) may face unique challenges in sustaining good adherence to HAART and treatment outcomes [1,2] while the potential for onward displacement presents a risk of pharmacy defaulting and treatment interruptions. An expectation of difficulty has clouded efforts to provide HAART in these settings [3]. Forcibly displaced populations consist mainly of refugees and IDPs, however, definitions are often confused. According to the Convention relating to the Status of Refugees, a refugee is a person who:

*"…owing to well-founded fear of being persecuted for reasons of race, religion, nationality, membership of a particular social group or political opinion, is outside the country of his nationality and is unable, or owing to such fear, is unwilling to avail himself of the protection of that country; or who, not having a nationality and being outside the country of his former habitual residence as a result of such events, is unable or, owing to such fear, is unwilling to return to it *[4]."

The Inter-agency Standing Committee defines IDPs as:

*"…persons or groups of persons who have been forced or obliged to flee or to leave their homes or places of habitual residence, in particular as a result of or in order to avoid the effects of armed conflict, situations of generalized violence, violations of human rights or natural or human-made disasters, and who have not crossed an internationally recognized State border *[5]."

“Conflict-affected persons” are defined as persons residing in active or recent conflict zones or in a post-conflict setting [6-8]. Around the world, some 1.5 billion people live in countries affected by violent conflict, 14.7 million people are internally displaced and 10.6 million are refugees. Of refugees, 68% had been living in exile for ≥5 years in “protracted situations” [9,10]. The primary aim of this review was to aggregate the available evidence on adherence to HAART and treatment outcomes in refugees, IDPs and conflict-affected persons, to assess the quality of work undertaken so far and to suggest future research needs.

## Methods

A systematic review of the published literature was conducted between 20 December 2011 and 20 January 2012 using a four step procedure. First, a search of the Cochrane Database of Systematic Reviews yielded no previous reviews. Second, we applied our search strategy to the Medline, Embase, and Global Health databases (including conference abstracts) using the Ovid platform. The search strategy incorporated five themes: “HIV”, “antiretroviral therapy”, “adherence”, “outcomes”, and “forced migration.” Key words were combined with medical subject headings (MeSH) to assess synonyms applicable to each theme. The terms “refugee”, “internally-displaced”, “conflict-affected”, and “forced migration” were used to search the forced migration theme. Table 1 presents a complete list of key words and MeSH terms used. As adherence results are often reported in papers where the primary aim is to report on clinical or treatment outcomes, “disease” and “treatment” themes were combined using the “OR” operator to create a broad pool which could be cross-referenced with the forced migration theme using the "AND" operator (Additional file 1: Table S1). Searches were limited to studies in English reported from 1995 onwards. Third, a backwards citation search was performed using the Web of Science “times cited” feature that identified all work cited by any previously identified report in the health science databases. Lastly, a check for sources that may have been posted online but omitted from health science databases was made by searching ReliefWeb and Médecins Sans Frontières (MSF) websites and three experts were consulted for additional sources. On the ReliefWeb and MSF-UK websites, we used the expressions “antiretroviral and refugee” and “antiretroviral and IDP.” On ReliefWeb, we searched under the “Analysis”, “Assessment”, “Evaluation”, “Situation Report”, “UN document” and “Other” content categories. Abstracts of papers retrieved from all the above steps that were not editorials, commentaries, case reports or had irrelevant titles were subjected to a full-text review. Both qualitative and quantitative studies of adult populations were included in the final dataset if they presented relevant primary data, secondary analyses on adherence to HAART or treatment outcomes, and included adult conflict-affected or forcibly displaced populations. We extracted and presented basic study information and data related to adherence and treatment outcomes. PRISMA guidelines were followed in the reporting of this review [11].

**Table 1:** Descriptions of quantitative studies included in the systematic review

**Location; study period [Ref]**	**Study type**	**Population [comparison group]**	**Time on HAART**	**Relevant reported HAART adherence outcome**	**Relevant reported treatment outcome**	**Adjusted analysis (outcome; factors associated with outcome with p<0.05)**
Lacor Hospital, Gulu District, Uganda; June 2005-Jan 2008 [12]	Prospective cohort study by structured questionnaire	IDPs, n=1625; 14% residing in IDP camps, 86% residing in outlying areas; >14 years-old	Cumulative patient-years follow-up=1981	Composite of pharmacy monitored drug possession ratio, pharmacy refill records, and 3-day self-reported recall by patients or caregivers:	Mortality incidence=3.48 (95% CI 2.66-4.31) per 100 person-years, log rank p-value<0.01;	Lower all-cause mortality:-Sex, female vs. male (HR=0.7 95% CI 0.55-0.91, p=0.02)
		[No comparison group]		≥95% doses taken as prescribed=92.2%	Median CD4 change (IQR)= 0 (0-0)	-Baseline CD4 count, per 100 cell increase (HR=0.14, 95% CI 0.06-0.34, p<0.001)
Lacor Hospital, Gulu District, Uganda; Jan-Feb 2008 [13]	Cross-sectional survey by semi-structured questionnaire	IDPs, n=200; 29% residing in IDP camps, 71% residing in outlying areas; ≥18 years-old	≤12 months=33.0%13-24 months= 29.5%>24 months=37.5%	Mean 4-day self-reported adherence recall, ≥95% doses taken as prescribed= 99.5%	NA	<95% adherence:-First line vs. second line treatment [OR=22.22, 95% CI 1.48-333.33, p=0.03]
		[No comparison group]				-Staff were condemning, yes vs. no (OR=22.22, 95% CI 1.5-333.33, p=0.02)
Nyanza province, Kenya; Dec 2007-July 2008 [14]	Retrospective cohort study by review of demographic surveillance data	IDPs, n=28 (proportion on HAART unknown); rural; ≥5 years-old	Not known	NA	53% (28/53) HIV mortality in IDPs vs. 25% (235/936) HIV mortality in 2008 DSS residents, p<0.001	NA
		[IDP HIV mortality compared with prior DSS residents]				
Kinkala/Mindouli, Republic of Congo; May 2006-Dec 2007 [15]	Retrospective cohort study by chart review	Conflict-affected, n=222; rural; adults ≥15 years-old	Mean follow-up time on HAART=9 months	NA	Probabilities of survival-(n=129) at 6 months=0.94 95% CI 0.89-0.96	NA
		[No comparison group]			-(n=70) at 12 months=0.89 95% CI 0.82-0.93	
12 MSF programs; Oct 2003- [2]	Retrospective cohort study by chart review	Conflict-affected, n=2572; rural; adults ≥15 years-old [No comparison group]	Median follow-up time on HAART=11.8 months (IQR 3.9-22.7)	NA	-Median probability of survival at 12 months 0.89, 95% CI 0.88-0.91	NA
					-Proportion lost to follow-up 0.11, 95% CI 0.09-0.12	
					-Median 6-month CD4 gain=129 cells/mm^3^	
**Location; study period [Ref]**	**Study type**	**Population [comparison group]**	**Time on HAART**	**Relevant reported HAART adherence outcome**	**Relevant reported treatment outcome**	**Adjusted analysis (outcome; factors associated with outcome with p<0.05)**
MSF Project, Bukavu, Democratic Republic of the Congo; May 2002-Jan 2006 [16]	Retrospective cohort study by chart review	Conflict-affected, n=494; urban and outlying areas [Compared with 18 low-income setting cohorts in Africa, Asia, and South America and 12 high-income setting cohorts in Europe and North America]	Person-years follow-up=235	>95% pills taken as prescribed as of last clinic visit, measured by pill counts=99%	6-month median CD4 gain (IQR): 163 (82-232)12-month mortality (95% CI)=7.9% (3.6-12.1)	NA
Equatorial region of southern Sudan; July 2009-March 2010 [17]	Retrospective cohort study by chart review	Refugees and IDPs, n=159 (69% living in refugee camps, 12% internally-displaced at time of HAART start); rural; adults age-cut-off not reported [No comparison group]	64% on HAART for ≥6 months	>95% adherence by self-report over past month=88% (of those on HAART for ≥6 months)	NA	NA
Nazareth House, Johannesburg, South Africa; April 2004-March 2007 [18]	Retrospective cohort study by chart review	Foreigners, n=568 (% refugees or IDPs unknown); urban; age≥16 [Compared with local citizens (n=431) and persons of unknown citizenship (n=298)]	Median (IQR) person-years on HAART:	NA	Viral failure (ART cessation, patient death, viral load >1000 copies/mL, any decrease in CD4 from pre-ART levels):	Viral failure (ART cessation, patient death, viral load >1000 copies/mL, any decrease in CD4 from pre-ART levels):
			-Foreigners=0.5 (0.1-0.9)-Local citizens=0.6 (0.2-1.1)		-Foreigners=24%-Local citizens=42%	-Citizenship status, foreigner vs. local citizen (OR=0.45, 95% CI 0.23-0.87, p= 0.017)
			-Unknown citizenship=0.5 (0.2-1.1)		-Unknown=53%p-value (foreigners vs. local citizens) = 0.001	-Opportunistic infections, TB before ART vs. none (OR=2.5, 95% CI 1.4-4.5, p=0.002)
Coptic Hope Centre for Infectious Diseases, Nairobi, Kenya; December 2006-February 2007 [19]	Retrospective cohort by chart review	Local population, n=2,534; Kenyan residents; age ≥18 [Compared with n=2,167 one year earlier, before post-election violence]	Median duration on treatment (IQR) 19.5 months (9.8-28.5) Comparison group was “similar”	Proportion interrupting treatment (visiting pharmacy ≥48 hours after ARTs completed)	NA	-Odds of TI during PEV increased by 71% 95%CI 34 to 118]
				-16.1% in PEV group-10.2% in comparison group		-During post-election violence, odds of TI increased for men (OR=1.37, 95%CI 1.07 to 1.76, p=0.01) and clients travelling ≥ 3 hours to clinic (OR=1.86, 95% CI 1.28 to 2.71, p=0.001)
**Location; study period [Ref]**	**Study type**	**Population [comparison group]**	**Time on HAART**	**Relevant reported HAART adherence outcome**	**Relevant reported treatment outcome**	**Adjusted analysis (outcome; factors associated with outcome with p<0.05)**
Miriam Hospital, Providence, Rhode Island, USA; 2000–2006 [20]	Matched case-control study by retrospective chart review	Refugees, n=52 (29 started ART); non-refugees, n=52 (41 started ART); urban [Controls were non-refugees matched on gender]	NR	Adherence to scheduled appointments:-Refugees=75%-Non-refugees=86%, p=0.17-Initiation of HAART: Refugees=56%-Non-refugees=79% (OR=0.37, 95% CI 0.13-0.92, p=0.03)	Not reported	NA
Mangere Refugee Resettlement Centre, Auckland, New Zealand; June 1993-June 2004 [21]	Retrospective cohort study by chart review	Refugees from Africa and Asia, n=98 (n=60 started HAART); urban [No comparison group]	NR	NA	Undetectable viral load 1 year after HAART start= 61% (36/59)	NA
Boston Medical Centre, USA; June 2000-June 2001 [22]	Retrospective cohort study by chart review	Refugees, n=34, n=15 on HAART; urban [No comparison group]	NR	“Reported adherence with medications”= 87%	Undetectable viral load (not defined)=87%	NA
Southern Alberta, Canada; Jan 2001-Jan 2007 [23]	Retrospective cohort study by chart review	Sub-Saharan African, n=126 (68% refugees); Other foreign-born, n=72 (14% refugees) [Canadian-born, n=455]	NR	“Good adherence within foreign-born patients to HAART” (data not shown)	80% viral suppression (no comparison between groups reported)	NA
Miriam Hospital, Providence, Rhode Island, USA; 2000–2006 [24]	Retrospective cohort study by chart review	Pregnant, resettled refugee women, n=14; rural [No comparison group]	NR	Lost to follow-up=1/14 (7%)	Median viral load at time of pregnancy=3.36 log_10_ copies/mL	NA
					Median viral load at time of delivery=1.88 log_10_ copies/mL	
Great Lukole camp, Tanzania; Oct 2002-Sept 2004 [25]	Retrospective cohort study by chart review	Women delivering in camp, n=189 [No comparison group]	NA	Single dose nevirapine uptake at labour=98% (185/189) excluding repatriated women and 62% (185/301) including refusals and repatriations	NA	NA

NA (not applicable)=outcome not applicable/analysis not done; NR (not reported)=data not reported.

IDP=internally-displaced person; OR=odds ratio; HR=hazard ratio; CI=confidence interval; IQR=inter-quartile range; HAART=highly active antiretroviral therapy; ART=antiretroviral therapy; TB=tuberculosis; TI=treatment interruption.

## Results

**Figure 1:**
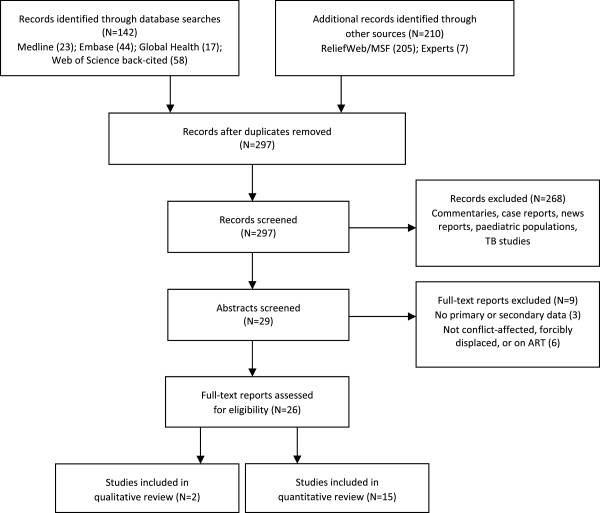
Study selection flowchart.

Figure 1 presents the outcome of the search: out of 297 reports retrieved using the search strategy, 17 reports conducted in 13 countries with 8,930 clients were retained for this review. Table 1 summarises the data extracted from selected reports. Three studies reported results for IDPs only. Kiboneka and colleagues [12] conducted a prospective cohort study to measure clinical and immunological outcomes of HAART clients in Gulu District, northern Uganda, the site of civil strife and conflict between the Ugandan government and the Lord’s Resistance Army guerrilla group. Adherence was measured by combining pharmacy monitoring, pharmacy refill records and patient self-report, and dichotomised at the ≥95% level. Over a median follow-up time of 13.7 months for clients with complete adherence data (n=1,521), 92.2% had ≥95% adherence. Among patients with <95% adherence, 9.3% died compared with 1.2% of patients with ≥95% adherence. In an adjusted analysis, mortality was less likely among women (Hazard ratio, HR=0.7, 95% confidence interval, 95%CI 0.55, 0.91; *p*=0.02) and clients with >200 CD4 at treatment start (HR=0.14, 95%CI 0.06, 0.34; *p*<0.001). A second cross-sectional study of IDPs in Gulu District reported a high (99.5%) mean self-reported four-day adherence to HAART [13]. In this study, clients who were on first-line therapy (Odds ratio, OR=22.22, 95%CI 1.53, 333.33; *p*=0.02) or who reported that clinic staff were “condemning” (OR=22.22, 95%CI 1.53, 333.33; *p*=0.02) were more likely to report non-adherence. A study of mortality among Kenyan IDPs in the post-election violence period of 2007–2008 found increased mortality in HIV-positive IDPs when compared with mortality during the same period among HIV-positive residents captured in the same Demographic Surveillance Survey catchment area prior to the violence [14].

We identified six studies that reported on conflict-affected, mixed refugee and IDP populations, or unspecified foreigners, including both refugees and asylum-seekers. O’Brien and colleagues [2], reporting on a pooled analysis of 12 MSF conflict and post-conflict HAART programs (n=2,572), found a median probability of survival at 12 months of 0.89 (95%CI 0.88, 0.91). Two further MSF studies of individual programs found good survival outcomes. In a post-conflict program in the Republic of Congo [15], the survival probability at 12 months was 0.89 (95%CI 0.82, 0.93). In a study from Bukavu, Democratic Republic of the Congo (DRC) during an active conflict involving the central government, insurgents and proxy armies from neighbouring states [16], optimal HAART adherence (defined as missing less than 5% of pills between clinic visits) measured by pill counts was attained by 99% of participants, although the limitations of this method in this setting were not specified. The conflict setting had higher 12-month mortality (7.9%, 95%CI 3.6, 12.1) than comparison settings, but the six-month median CD4 cell gain of 163 cells/mm^3^ compared favourably with cohorts from a resource-limited setting (106 cells/mm^3^) and a resource-rich setting (103 cells/mm^3^). In the Equatorial province of Sudan, Salami and colleagues [17] found that 88% of refugees and IDPs on HAART for ≥6 months self-reported ≥95% adherence. A South African study comparing self-identified foreigners with local citizens reported that foreigners were less than half as likely (OR=0.45, 95%CI 0.23, 0.87; *p*= 0.017) to have suffered viral failure, defined as ART cessation, any decrease in CD4 from pre-ART levels, a viral load of >1000 copies/mL, or death [18]. In a Kenyan study that used a pre-post design to investigate treatment interruptions (TI) (defined as the proportion visiting pharmacy ≥48 hours after ARTs completed), 16.1% experienced a TI during post-election violence compared with 10.2% in the comparison period [19]. For clients who overlapped between the two periods, the odds of TI were elevated by 71% during post-election violence (95%CI 34, 118, p<0.001]. Men (OR=1.37, 95%CI 1.07, 1.76; *p*=0.01) and clients travelling more than three hours to the clinic (OR=1.86, 95%CI 1.28, 2.71; *p*=0.001) also were more likely to experience a TI.

Four studies were conducted among HIV-positive refugees in high income settings. An HIV-positive refugee cohort in Rhode Island, USA, had lower odds of initiating HAART when compared with non-refugees (OR=0.37, 95%CI 0.13, 0.92; *p*=0.03) and had a lower attendance at scheduled appointments relative to non-refugees (75% v. 86%, *p*=0.17) [20]. In a New Zealand-based refugee cohort on HAART, 61% had an undetectable HIV viral load after one year of treatment [21]; a US study reported undetectable viral load in 87% of refugees receiving HAART [22]. In a Canadian study, 80% of a cohort consisting of native-born clients, refugees and other immigrants from Sub-Saharan African and elsewhere were virologically suppressed [23]. In this study, the rate of progression to new opportunistic infections or AIDS-defining events was higher among the former group (0.1 v. 0.06 events/1000 patient-days) while the mortality rate restricted to HIV-related deaths was higher among the latter group (0.8 v. 1.2 deaths/1000 patient-months).

Two studies dealt exclusively with pregnant mothers taking ART for prevention of mother-to-child transmission (PMTCT). The first study, conducted among a resettled group of refugees on HAART during pregnancy in Rhode Island, USA, reported a reduction in median viral load at the time of delivery [24]. A study conducted in a Tanzanian refugee camp reported a 98% (185/189) uptake of nevirapine at the time of delivery, but women who initially refused the medication or who were repatriated to their native countries prior to delivery were not included [25].

Two additional qualitative studies were also eligible for review. One study conducted in Teso, northern Uganda [26], identified security while attending clinics, food security, distance to health centres and access to health providers as the main concerns of clients and health workers in relation to HAART adherence. Respondents noted that food insecurity and single daily meals made multiple daily dosing a challenge during famines and floods. A second study from northern Uganda [27] reported on the impact of social networks on long-term provision of antiretrovirals. This study reported that community-based volunteers and health workers were effective in supporting adherence and the formation of social support groups, while social networks assisted in overcoming challenges that were independently related to displacement and stigma. Notably, but perhaps unsurprisingly, the study identified inadequate planning in the return phase of the forced displacement cycle as presenting significant challenges in patient monitoring, missed appointments, and loss to follow-up. Finally, in a qualitative sub-study (counted as a quantitative study for the purposes of Figure 1), Pyne-Mercier and colleagues [19] reported that 6/ 13 interviewed clients had been attacked by mobs or had their homes or businesses vandalised during post-election violence in Kenya. Lack of transport and inflated transport costs were identified as barriers to accessing treatment, while personal commitment and support from family and clinic social workers facilitated access to treatment.

## Discussion and conclusions

This review revealed a limited number of studies on adherence to HAART and treatment outcomes, however the outcomes observed in the reviewed studies showed promise for conflict-affected and forcibly displaced populations. The range of optimal adherence prevalence of 87–99.5% compared favourably with other settings. A meta-analysis of 84 observational studies reported a 62% average reporting rate of ≥90% adherence [28]. A second meta-analysis comparing resource-limited and resource-rich settings [29] reported that 55% of North American populations and 77% of sub-Saharan African populations achieved adequate adherence. Important factors negatively affecting adherence in sub-Saharan Africa included non-disclosure of HIV status to a loved one for fear of stigma, alcohol abuse, and difficulty following complex drug regimens. In a separate review of barriers to adherence, pooled results from ten quantitative studies in developing countries identified financial constraints (52%, 95%CI 16, 88) and forgetfulness (36%, 95%CI 19, 55) as major barriers [30]. A review of African studies [31] reported that 68-99% of patients were ≥95% adherent but found no studies documenting the use of formal adherence intervention programs. The present review identified one study [13] that compared conflict-affected or forcibly displaced groups to a local host community or that assessed potential barriers to, and facilitators of adherence and/or treatment outcomes. Garang and colleagues found no significant differences in adherence between IDPs and non-IDPs (99.6% v. 99.5%) and reported that being on first-line treatment and clients’ perceived condemnation by medical staff reduced the odds of optimal adherence. Barriers to adherence in this study included depression after losing a child, forgetfulness, travelling, and not refilling medications on schedule.

We located one study that specifically studied treatment interruptions [19]. Understanding the prevalence and consequences of treatment interruptions is highly relevant for conflict-affected and forcibly displaced groups given their previous displacement history and the potential for onwards travel including resettlement or repatriation after initiation of therapy. Studies in other population groups that investigated interruptions as therapeutic alternatives to continuous therapy such as intentional treatment holidays and unintentional interruptions have found harmful results. Initial concerns about long-term safety and reports of a lack of improved virological response in trials of structured interruptions [32,33] were confirmed by the Strategies for Management of Antiretroviral Therapy (SMART) study, which found that CD4-guided episodic therapy increases the risk of opportunistic disease or death in relation to continuous treatment [34]. A review of unstructured TIs found an increased risk of opportunistic infections, virological failure, drug resistance, poor immunological recovery and death [35]. Future studies on forcibly displaced and conflict-affected groups should facilitate the monitoring of TIs by combining data from facility-based pharmacy records and mobile phone follow-up contact with clients that confirm TIs when clients fail to report for routine pharmacy refill appointments.

The studies included in this review were conducted in a variety of contexts including camps, rural, and urban areas in low-income settings and urban areas in high-income settings. Although the studies did not describe the specific features of the clinics where HAART was delivered, these were likely variable in relation to the type of institutional provider, which ranged from publicly-run hospitals to non-governmental organisations, and their respective resource levels. Given the importance of context for outcomes, the variation in settings may have affected the reported findings and merit further study. The majority (14/15) of the reviewed quantitative studies were facility-based. When clients present at new treatment facilities, HIV testing and counselling is routinely administered where indicated and HAART is initiated according to national guidelines, regardless of treatment history. With the exception of the MSF studies where no previous HIV testing had taken place, the question of whether treatment start at the study facility was equivalent to treatment initiation, or if treatment had been started elsewhere prior to onwards displacement and arrival at the study facility, was not verifiable. Establishing the date and location of HAART initiation is challenging in any setting where medical records are not routinely shared and client recall of their complete treatment history may be compromised. Where possible, investigations that attempt to address these shortcomings will be useful for estimating the effect of forced displacement on adherence and treatment outcomes and for correctly interpreting findings. Moreover, the categorisation of displaced persons presents additional challenges; definitions may affect the network of providers, the availability of particular services, and the extent of co-payments (if any). For example, in non-refugee camp settings a lack of documentation or xenophobic attitudes may present obstacles to accessing key services including HIV counselling, testing, and ART. To facilitate generalisability to similar settings and population groups, future studies should be mindful of these categorizations and their impact on outcomes.

There were some limitations to this review. Although we searched health databases and grey literature, it is possible that relevant studies were omitted; we limited our search to reports published in English. To minimise this risk of exclusion, we used a backwards citation search and expert consultation. Most identified studies used a single adherence indicator, which suggested a possibility of measurement bias. AlthoughÂ Â there is noÂ widely accepted standard for measuring adherence, self-report andÂ pharmacy refills are the most commonly used instruments in resource-limited settings [36,37]. Triangulation is one way to enhance confidence in measurement validity, especially in challenging multi-linguistic or complex emergency settings. Guidelines developed by an International Association of Physicians in AIDS Care Panel recommended routine use of both self-reports and pharmacy refill measures [38]. Where possible, multiple adherence measurements should be used, especially when more objective measures such as medication event monitoring systems (MEMS) are not feasible and biomarkers are not available from medical records or are too difficult or expensive to collect.

The geographic breadth of quantitative studies was limited: 53% (8/15) of the studies were conducted with asylum-based refugees and IDPs in African settings. Notably, only one study dealt with documented refugees in low-income settings [17]. The limited number of studies, small sample sizes (five included <100 clients), lack of comparison groups and varied outcomes and indicators suggest that estimates may have suffered from selection and response biases. We did not undertake a meta-analysis due to the substantial differences between client groups, methods, and outcomes across studies. Despite these difficulties, the reviewed studies were designed around local circumstances: samples were either limited by the absolute number of clients with available records to review, the study was facility-based and only had access to a limited pool of clients, or an evaluation of adherence and treatment outcomes was not the primary aim. Response bias was likely to have been less of a concern in the study by Kiboneka and colleagues [12], where a comprehensive adherence assessment of all HIV-positive clients attending one hospital was undertaken. However, the risk that the small number of studies available for review were conducted in settings more suitable for research, for example where data existed in a form particularly conducive to chart reviews, may have biased the findings towards better outcomes.

If HAART is to be scaled-up in conflict-affected and forcibly displaced clients, studies designed to assess adherence and treatment outcomes will be critical for optimising treatment outcomes and preventing drug resistance associated with widespread distribution of medications, the use of less tolerated regimens, restricted virological monitoring, and the potential for inconsistent drug supply [39]. The World Health Organisation’s (WHO) public health strategy for mitigating drug resistance recommends providing highly effective first-line regimens, prescribing previously unused drug classes when switching after first-line treatment failure, reserving the drugs that are least likely to provoke resistance for patients whose first-line treatments are no longer effective, and administering regimens that encourage adherence [40]. For forcibly-displaced and conflict-affected clients, these principles raise important questions. What is the most effective first-line regimen for these settings [41]? When clients are displaced and have a poor knowledge of their treatment history, which HAART regimen should be used? Are HIV-positive individuals who were started on HAART prior to displacement identified quickly in the host setting, linked to care in a timely manner, and succeeding on treatment? Are best practices correctly implemented prior to voluntary repatriation or resettlement to a third country? Which factors, regimen-related or otherwise, encourage good adherence? Recent intervention studies in resource-limited settings have shown that counselling services and mobile phone-based reminders helped to maintain adherence and viral suppression [42,43]. Although trials have not been conducted among conflict-affected and forcibly-displaced populations, a useful basis for an intervention consisting of aÂ 7-step support package andÂ tailored to the needs of migrants, refugees,Â and asylum-seekersÂ was delivered by MSF in Musina. This report, published after our review period, found that 92% (95%CI 75.2, 97) of clients were virologically suppressed (<400 copies/mL) at 12 months after receiving this intervention, which included a patient-held record (“health passport”), an alternative treatment site road map, anticipation of travel at regular clinic visits, a safe travel pack (including buffer stock of ARVs, a washout regimen, and a transfer letter), migrant-adapted treatment counselling, a questionnaire for returning patients and migrant-adapted monitoring of retention in care [44].

In summary, the limited evidence from the small series of studies available for this review suggests that HAART adherence and treatment outcomes among conflict-affected and forcibly displaced adults may be as good as outcomes attained in unaffected population groups. Future research should consider stronger study designs that address TIs throughout the displacement cycle, more geographic variation, the use of a systematic, replicable, and triangulated approach to adherence monitoring, and the design and testing of interventions to improve adherence and treatment outcomes. Given that refugees in asylum countries tend to remain for an average of 17 years [45], there is a strong national interest and humanitarian rationale for ensuring universal access to HIV treatment and care, promoting optimal outcomes among all vulnerable groups and developing a consensus approach to achieving these goals [46]. For effective HAART scale-up in conflict-affected and forcibly displaced clients, assessing adherence and treatment outcomes will be critical for promoting viral suppression, preventing drug resistance and reducing onward transmission.

## Abbreviations

ART: Antiretroviral therapy; cART: Combination antiretroviral therapy; CI: Confidence interval; DRC: Democratic Republic of the Congo; HAART: Highly active antiretroviral therapy; HR: Hazard ratio; HIV: Human immunodeficiency virus; IDP: Internally displaced person; MSF: Médecins Sans Frontières; PEV: Post-election violence; PMTCT: Prevention of mother-to-child transmission; OR: Odds ratio; TI: Treatment interruption; UNHCR: United Nations High Commissioner for Refugees.

## Competing interests

The authors declare that they have no competing interests.

## Author contributions

JBM designed and implemented the search strategy and wrote the first draft of the manuscript. DAR reviewed the search strategy, supported interpretation of findings, and commented on the manuscript. MS and PS supported the interpretation findings and commented on the manuscript. All authors edited the final draft for intellectual content, and approved the final manuscript.

## Supplementary Material

Additional file 1 **Table S1.** Systematic review search strategy used in MEDLINE*. Click here for file
