# Supplementary material for: Adherence to antiretroviral therapy and treatment outcomes among conflict-affected and forcibly displaced populations: a systematic review
Source: Confl Health. 2012 Oct 31;6:9. doi: 10.1186/1752-1505-6-9 (PMC3533728; doi:10.1186/1752-1505-6-9)
Supplement: Additional file 1 — Table S1. Systematic review search strategy used in MEDLINE*. [file 1752-1505-6-9-S1.docx]

| Supp. Table i: Systematic review search strategy used in MEDLINE* | | |
| --- | --- | --- |
| Step | Search theme | Key word(s) and MeSH term(s) |
| 1 | Forced migration | [refugee*] or [forced migra*] or [internally-displaced] or [conflict-affected] or [exp Refugees/] |
| 2 | HIV disease | [HIV] or [AIDS] or [exp HIV/] or [exp HIV-2/] or [exp HIV-1/] |
| 3 | Adherence | [adherence] or [compliance] or [exp Medication Adherence/ ] or [exp Patient Compliance/] or [treatment interruption*] |
| 4 | Antiretroviral therapy | [antiretroviral therapy] or [HAART] or [ART] or[ cART] or [exp Anti-HIV Agents/] or [exp Antiretroviral Therapy, Highly Active/] |
| 5 | Treatment outcomes | [treatment outcome*] or [exp Treatment Outcome/] or [exp RNA, Viral/ ] or [exp Viral Load/] or [cd4] or [exp Antigens, CD4/] or [viral load] |
| 6 | Adherence AND Treatment outcomes | [adherence] or [compliance] or [exp Medication Adherence/ ] or [exp Patient Compliance/] or [treatment interruption*] AND [antiretroviral therapy] or [HAART] or [ART] or[ cART] or [exp Anti-HIV Agents/] or [exp Antiretroviral Therapy, Highly Active/] |
| 7 | Adherence AND HIV disease | [adherence] or [compliance] or [exp Medication Adherence/ ] or [exp Patient Compliance/] or [treatment interruption*] AND [HIV] or [AIDS] or [exp HIV/] or [exp HIV-2/] or [exp HIV-1/] |
| 8 | Antiretroviral therapy AND Treatment outcomes | [antiretroviral therapy or HAART or ART or cART] OR [exp Anti-HIV Agents/ OR exp Antiretroviral Therapy, Highly Active/] AND [treatment outcome*] or [exp Treatment Outcome/] or [exp RNA, Viral/ ] or [exp Viral Load/] or [cd4] or [exp Antigens, CD4/] or [viral load] |
| 9 | HIV disease AND Treatment outcomes | [HIV] or [AIDS] or [exp HIV/] or [exp HIV-2/] or [exp HIV-1/] AND [treatment outcome*] or [exp Treatment Outcome/] or [exp RNA, Viral/ ] or [exp Viral Load/] or [cd4] or [exp Antigens, CD4/] or [viral load] |
| 10 | Forced migration AND all themes | [Forced migration] AND [(Adherence AND Treatment outcomes) or (Adherence AND HIV disease) or (Antiretroviral therapy AND Treatment outcomes) or (HIV disease AND Treatment outcomes)] |
| 11 | Limits | Limited to English studies from 1995 onwards |
| “or” preceded “AND” in order of operations | | |
| exp=explode a MeSH term | | |
| “ / ”=included all MeSH subheadings | | |
| *similar strategies were used in Global Health and Embase databases, with the exception of differences in MeSH terms | | |
